# Left atrial contractile strain is the independent predictor of LV remodeling after ST-segment elevation myocardial infarction

**DOI:** 10.1186/1532-429X-17-S1-P151

**Published:** 2015-02-03

**Authors:** Krzysztof P Karwat, Marek Tomala, Karol Miszalski-Jamka, Wojciech Mazur, Dean J Kereiakes, Jadwiga Nessler, Krzysztof Zmudka, Tomasz Miszalski-Jamka

**Affiliations:** Department of Coronary Disease and Heart Failure, John Paul II Hospital, Kraków, Poland; Department of Haemodynamics and Angiocardiography, John Paul II Hospital, Kraków, Poland; Department of Cardiology, Congenital Heart Disease and Electrotherapy, Silesian Center for Heart Disease, Zabrze, Poland; The Christ Hospital Heart and Vascular Center, The Lindner Center for Research and Education, Cincinatti, OH USA; Department of Radiology and Imaging Diagnostics, John Paul II Hospital, Kraków, Poland; Center for Heart Disease, Clinical Military Hospital, Wrocław, Poland

## Background

There is growing evidence on the value of left atrial function assessment in cardiac magnetic resonance (CMR) in patients with heart failure. However, there is paucity of data regarding the value of left atrial function in CMR to predict left ventricular remodeling in patients with ST-segment elevation myocardial infarction (STEMI).

## Methods

One hundred one (79 males, 22 females, mean age 58.3±11.5 years) patients, who underwent CMR 3-5 days and 6 months after first STEMI treated with primary percutaneous coronary intervention, were enrolled. Cine, T2-weighted STIR and late gadolinium enhancement (LGE) CMR images were assessed off-line using dedicated software (QMass 7.5, Medis, Leiden, The Netherlands). Feature tracking cine-sequence based left atrial strain were measured using Diogenes CMR (TomTec Imaging Systems, Munich, Germany) in 2- and 4-chamber views. Segmental strain values were averaged from two views to obtain average reservoir, conduit and contractile strain. Logistic regression analysis was performed in stepwise forward fashion to determine CMR predictors of LV remodeling. The outcomes were expressed by odds ratio (OR) with corresponding 95% confidence interval (CI).

## Results

52 subjects had anterior and 49 inferior STEMI. LV remodeling (increase in LV end-systolic volume by 15% in 6 months after STEMI) was present in 29 subjects. Comparing subjects with and without LV remodeling the former tended to have larger extent of LVLGE (27.9±14.3 versus 22.2±13.8% of LV myocardium; p=0.07), larger extent of microvascular obstruction (MO) within LGE (4.3±5.3 versus 2.2±3.1% of LV myocardium; p=0.02), higher maximal levels of troponin I (147.1±122.1 versus 91.2±72.4 ng/ml; p=0.006), CPK-MB (691±407 versus 363±237 U/l; p<0.001), CPK (4943±2968 versus 2733±1936 U/l; p<0.001), and had lower values of average left atrial reservoir strain (14.6±5.3 versus 25.1±10.0%; p<0.001), left atrial conduit strain (6.3±4.3 versus 9.1±5.4%; p=0.02) and left atrial contractile strain (8.3±2.8 versus 16.0±6.2%; p<0.001). The univariative predictors of LV remodeling were the extent of MO within LGE (OR=1.14; 95%CI:1.01-1.27; p=0.02), maximal levels of troponin I (OR=1.006; 95%CI:1.002-1.011; p=0.007), CPK-MB (OR=1.004; 95%CI:1.002-1.010; p<0.001), CPK (OR=1.0004; 95%CI:1.0002-1.0006; p<0.001), and average left atrial reservoir strain (OR=0.80; 95%CI:0.72-0.88; p<0.001), left atrial conduit strain (OR=0.86; 95%CI:0.77-0.98; p=0.01) and left atrial contractile strain (OR=0.62; 95%CI:0.50-0.77; p<0.001). Of those in multivariative analysis the independent predictors were CPK (OR=1.0003; 95%CI:1.0000-1.0006; p=0.048) and left atrial contractile strain (OR=0.65; 95%CI:0.53-0.80; p<0.001).

## Conclusions

LV remodeling is frequent after STEMI. The average left atrial contractile strain is the independent predictor of LV remodeling in 6 months observation.

## Funding

N/A.

